# A Novel Inhibitory Mechanism of Mitochondrion-Dependent Apoptosis by a Herpesviral Protein

**DOI:** 10.1371/journal.ppat.0030174

**Published:** 2007-12-07

**Authors:** Pinghui Feng, Chengyu Liang, Young C Shin, Xiaofei E, Weijun Zhang, Robyn Gravel, Ting-ting Wu, Ren Sun, Edward Usherwood, Jae U Jung

**Affiliations:** 1 Department of Microbiology and Molecular Genetics and Tumor Virology Division, New England Primate Research Center, Harvard Medical School, Southborough, Massachusetts, United States of America; 2 Department of Microbiology, University of Texas Southwestern Medical Center, Dallas, Texas, United States of America; 3 Department of Microbiology and Immunology, Dartmouth Medical School, Hanover, New Hampshire, United States of America; 4 Department of Molecular and Medical Pharmacology, University of California at Los Angeles, Los Angeles, California, United States of America; University of California San Francisco, United States of America

## Abstract

Upon viral infection, cells undergo apoptosis as a defense against viral replication. Viruses, in turn, have evolved elaborate mechanisms to subvert apoptotic processes. Here, we report that a novel viral mitochondrial anti-apoptotic protein (vMAP) of murine γ-herpesvirus 68 (γHV-68) interacts with Bcl-2 and voltage-dependent anion channel 1 (VDAC1) in a genetically separable manner. The N-terminal region of vMAP interacted with Bcl-2, and this interaction markedly increased not only Bcl-2 recruitment to mitochondria but also its avidity for BH3-only pro-apoptotic proteins, thereby suppressing Bax mitochondrial translocation and activation. In addition, the central and C-terminal hydrophobic regions of vMAP interacted with VDAC1. Consequently, these interactions resulted in the effective inhibition of cytochrome c release, leading to the comprehensive inhibition of mitochondrion-mediated apoptosis. Finally, vMAP gene was required for efficient γHV-68 lytic replication in normal cells, but not in mitochondrial apoptosis-deficient cells. These results demonstrate that γHV-68 vMAP independently targets two important regulators of mitochondrial apoptosis-mediated intracellular innate immunity, allowing efficient viral lytic replication.

## Author Summary

Apoptosis is a conserved cell death program that contributes to restriction of viral replication and elimination of infected cells. Whether triggered via internal inducers such as DNA damage or via external stimuli such as engagement of the death receptor, apoptosis takes place through a cascade of regulated internal proteolytic digestion, resulting in a collapse of cellular infrastructure, mitochondrial potential, genomic fidelity, and cell membrane integrity. Indeed, apoptosis represents a predominant form of virally infected cell demise. In response, viruses have evolved numerous ways of circumventing this host-cell apoptosis. Most of the DNA viruses including murine γ-herpesvirus 68 (γHV-68) are genetically equipped with anti-apoptotic ability to ensure viral replication and propagation. The authors have identified a new viral mitochondrial protein (vMAP) of γHV-68 that interacts with Bcl-2 and voltage-dependent anion channel 1 (VDAC1) in a genetically separable manner. These interactions markedly suppress Bax mitochondrial translocation and activation and inhibit cytochrome c release, leading to the comprehensive inhibition of mitochondrion-mediated apoptosis. The authors also demonstrate that vMAP gene is required for efficient γHV-68 lytic replication in normal cells, but not in mitochondrial apoptosis-deficient cells. These findings are entirely novel and significantly advance our understanding of how virus escapes host intracellular apoptosis-mediated innate immunity.

## Introduction

Apoptosis, or programmed cell death, has a key role in a variety of eukaryotic biological processes such as development and immune responses. Whether initiated by receptor ligation at the cell surface or through signal transduction from intracellular organelles, both caspase-dependent and -independent apoptotic pathways degrade cellular components, leading to the complete dismantling of targeted cells. Particularly, mitochondria serve as gatekeepers for the life-or-death decision, conveying apoptotic signals by releasing death-promoting factors (e.g., cytochrome c, apoptosis-inducing factor, and DIABLO/Smac from the intermembrane space [1–4]).

Recent studies have largely elucidated the complex mechanism that eukaryotes have evolved to regulate the permeability of the mitochondrial outer membrane during apoptosis, particularly with regard to Bcl-2 homology (BH) family proteins [5–7]. Bcl-2 family members are classified as either anti-apoptotic (e.g., Bcl-2, Bcl-x_L_, Bcl-w, and A1) or pro-apoptotic, the latter of which can be further divided into the multi-BH domain and BH3-only subgroups. Similar to anti-apoptotic proteins, the multi-BH domain members, such as Bax and Bak, adopt a globular fold consisting of up to nine a-helices that form an extended hydrophobic cleft on the surface [8]. This cleft serves as an authentic BH3-binding site that mediates their translocation and oligomerization within the mitochondrial outer membrane and, ultimately, mitochondrial permeabilization [6,9].

Additional regulation of the mitochondrion-dependent apoptosis is mediated by the permeability transition pore (PTP), a complex composed of the voltage-dependent anion channel (VDAC) and adenine nucleotide translocator (ANT). VDAC is located in the mitochondrial outer membrane, and its transmembrane can form a barrel with a pore size consistent with the estimated open-channel diameter to allow the escape of cytochrome c. A direct interaction between Bcl-2 family members and VDAC may control cytochrome c permeation across membranes [7,10]. Alternatively, VDAC-mediated closure of the PTP supercomplex may prevent ATP/ADP exchange across the membrane, which ultimately results in outer membrane permeabilization and subsequent release of pro-apoptotic factors from the intermembrane space; this, in turn, triggers apoptosis in the cytoplasm or nucleus [10]. Although current studies favor the latter model, these hypotheses may not be mutually exclusive in defining the nature of the PTP complex.

Apoptosis is part of host innate immunity that eliminates the propagation of intracellular pathogens such as viruses. As a countermeasure, viruses have evolved to encode numerous open reading frames (ORFs) to circumvent cellular apoptotic pathways. Herpesviruses, particularly γ-herpesviruses, including human Epstein-Barr virus, Kaposi sarcoma–associated herpesvirus, and murine γ-herpesvirus 68 (γHV-68), provide unique models for dissecting the molecular mechanisms of apoptotic regulation. A common aspect of these γ-herpesviruses is that they encode viral homologs of Bcl-2, designated vBcl-2, and these vBcl-2 genes effectively prevent cells from undergoing apoptosis upon various stress responses [11,12]. vBcl-2 has been speculated to serve a vital role in the virus lifecycle by inhibiting the premature apoptotic death of host cells during acute replication, allowing completion of viral replication cycle and favoring the spread of progeny virus. However, the previous studies have found that γHV-68 vBcl-2 has no role during acute infection but its activity is critical specifically to latent infection [13–15]. Thus, to identify viral protein that evades host apoptosis-mediated innate immunity during acute infection, we searched for mitochondrial proteins within the γHV-68 genome. We report that viral mitochondrial anti-apoptosis protein (vMAP) specifically interacts with cellular Bcl-2/Bcl-x_L_ and VDAC and that these interactions effectively dampen host apoptotic processes, which ultimately contributes to efficient lytic replication in culture.

## Results

### The γHV-68 vMAP Gene Encodes a Mitochondrial Anti-Apoptotic Protein

**Figure 1:**
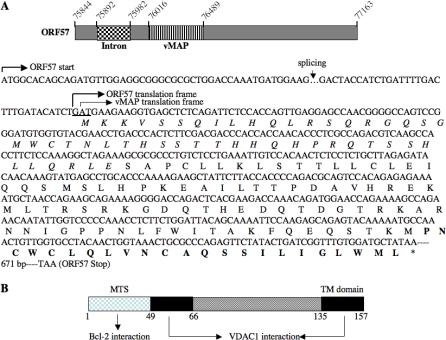
Genomic Localization and Protein Organization of γHV-68 vMAP (A) vMAP is encoded within the ORF57. (Top diagram) The vMAP genomic localization is shown within the ORF57 sequence. Numbers indicate the nucleotide position according to Entrez accession number U97553. The grid box represents a 90–base pair intron of ORF57 and the box with vertical lines represents vMAP (M8). (Bottom diagram, sequence) The first ATG denotes the start codon for ORF57 (arrow on the left). The coding sequence of vMAP is shown in reference to ORF57 and its amino acid sequence is listed below. The translation of vMAP has a +1 shift to ORF57 frame; thus, the vMAP amino acid sequence is completely different from the ORF57 amino acid sequence. The mitochondrion-targeting sequence (MTS) is italicized and the putative transmembrane (TM) domain is indicated by bold letters. An arrow (↓) over a gap (dots) indicates the splicing site where a 90–base pair of intron is removed [47]. *, stop codon. (B) vMAP protein organization. The N terminal sequence (aa 1–49) serves as a mitochondrial targeting sequence (MTS) as well as a Bcl-2-interacting region. Both the internal hydrophobic region (aa 50–66) and the putative transmembrane (TM) domain (aa 135–157) interact with VDAC1.

**Figure 2:**
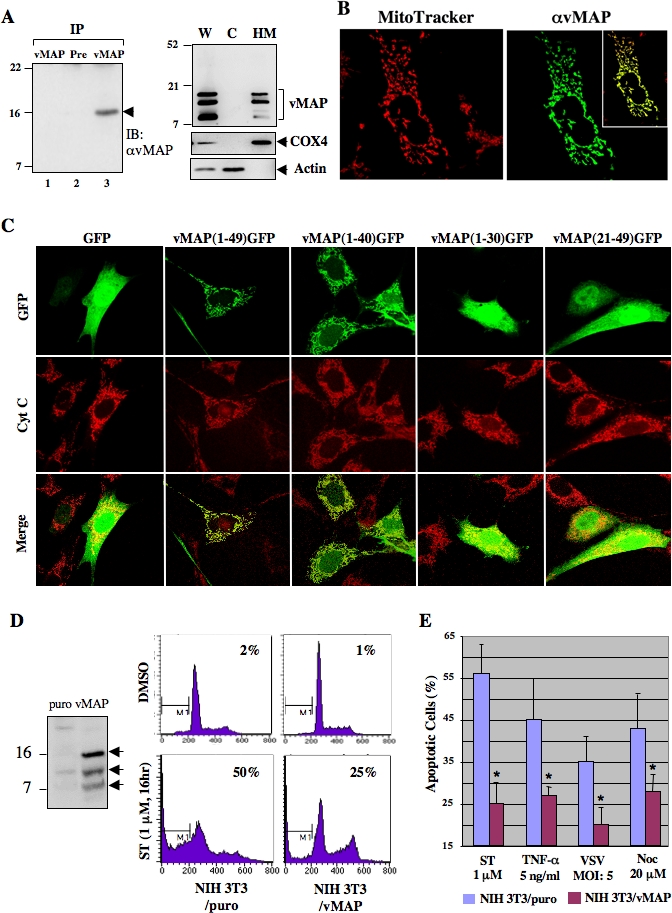
γHV-68 vMAP Encodes a Mitochondrial Protein (A) Identification of vMAP protein. (Left panel) γHV-68-infected (lane 2 and 3) or mock-infected NIH3T3 cells (lane 1) were harvested at 16 h post-infection. Post-centrifuged lysates in CHAPS buffer were precipitated with pre-immune serum (lane 2) or anti-vMAP serum (lanes 1 and 3), followed by immunoblotting (IB) with anti-vMAP serum. The arrowhead indicates vMAP. (Right panel) Subcellular fractionation of vMAP in 293T cells at 36 h post-transfection, as shown by immunoblotting with antibodies to vMAP, COX4, or actin. W, whole cell lysate; C, cytosolic; HM, mitochondrion-enriched heavy membrane. (B) vMAP localizes to the mitochondrion. Post-transfection with pcDNA5-vMAP vector, COS-1 cells were stained with MitoTracker and fixed for anti-vMAP immunostaining. The box inside of the right panel shows the merged image of MitoTracker and anti-vMAP immunostaining. (C) Identification of the vMAP MTS. Either GFP alone or various vMAP N-terminal sequences fused to GFP were expressed in NIH3T3 cells. Mitochondria were stained with anti–cytochrome c antibody (red). Pictures represent more than 85% of cells analyzed by fluorescent microscopy. (D) vMAP inhibits apoptosis. NIH3T3/puro and NIH3T3/vMAP cells were treated with DMSO or ST (1 μM) for 16 h and stained with PI, followed by flow cytometry analysis. Data are from one of three replicate experiments. vMAP (indicated by arrows) expression was shown by immunoblotting on the left. (E) vMAP inhibits apoptosis initiated by various apoptogenic stimuli. NIH3T3/puro and NIH3T3/vMAP cells were treated with various agents (ST, TNF-α/cycloheximide, vesicular stomatitis [VSV] infection for 16 h, or nocodazole [Noc] for 36 h), and sub-G1 cells were quantified as described in (D). Data represent result of three independent experiments and error bars indicate standard deviation with (*) *p* < 0.05 relative to control (puro) as calculated by Student's *t*-test.

To test the role of the mitochondrion-dependent apoptosis in γHV-68 replication, wild-type (wt) and Bax^−/−^Bak^−/−^ double knockout (DKO) murine embryonic fibroblasts (MEFs) were infected with a GFP-containing γHV-68 (γHV-68ΔK3-GFP). DKO MEFs supported γHV-68ΔK3-GFP replication with accelerated kinetics as compared with wt MEFs: the virus titer in DKO MEFs was 50–100 times higher than that in wt MEFs (Figure S1A and S1B). Furthermore, as previously shown [16], DKO MEFs displayed markedly reduced cell death upon γHV-68 replication than did wt MEF (Figure S1C and S1D). These results indicate that mitochondrion-mediated apoptosis plays a negative role in γHV-68 lytic replication, suggesting that γHV-68 needs to deregulate this pathway to maximize its propagation. To search for the potential mitochondrial protein(s) encoded by γHV-68, we employed two computer programs, MITOProt (http://mips.gsf.de/cgi-bin/proj/medgen/mitofilter) and PSORT (http://psort.nibb.ac.jp/), which assess the likelihood of each candidate viral gene product to be targeted to the mitochondrion. This survey identified an M8 gene product [17] that we have named vMAP. vMAP is present in the second exon of ORF57 and shares the identical nucleotide sequence with ORF57 (Figure 1A). However, vMAP has a +1 shift in reference to ORF57 frame, thus encoding a polypeptide of distinct amino acid sequence from ORF57. vMAP contains 157 amino acids, with a predicted mitochondrial targeting sequence (MTS) at its N-terminus and a putative transmembrane domain at its C-terminus (Figure 1B). vMAP protein was readily detected during γHV-68 lytic replication with an apparent molecular weight of 16 kDa (Figure 2A, left panel). However, we failed to detect vMAP protein in γHV-68 latently infected S11 cells under the same conditions (unpublished data). When vMAP expression vector was transfected into NIH3T3 cells, however, vMAP migrated as 7-, 14-, and 16-kDa species (Figure 2A, right panel). Mutational analyses indicated that the 7- and 14-kDa proteins were derived from translational initiation at the third (Met_70_) and second (Met_21_) internal initiation codon, respectively (unpublished data). Intracellular fractionation demonstrated that vMAP was present exclusively in the mitochondrion-enriched heavy membrane (HM) fraction (Figure 2A, right panel). The position and integrity of the fraction was confirmed by the presence of the mitochondrial resident protein COX4. Confocal immunofluorescence microscopy also showed that vMAP was present in the cytoplasm extensively colocalized with MitoTracker, a dye that specifically labels mitochondria in living cells, and with Hsp60 and cytochrome c mitochondrial resident proteins (Figures 2B, S2A, and S2C). GFP fusions containing the N-terminal region of vMAP were constructed to define the MTS of vMAP. The N-terminal 40 residues of vMAP were sufficient to target GFP to mitochondria (Figure 2C). Interestingly, this N-terminal sequence of vMAP is predicted to contain α-helical structure, followed by a stretch of positively charged residues that are the potential MTS motif (Figure 1A). In fact, deletion mutations within this motif considerably impaired the mitochondrial localization of GFP fusions containing vMAP N-terminal sequences in confocal microscopy and intracellular fractionation (Figures 2C and S2B). Of note, the vMAP(1–30)-GFP and vMAP(21–49)-GFP also showed the nuclear localization, which was likely contributed by GFP fusion (Figure 2C). These results indicate that the N-terminal 40 residues are sufficient for mitochondrial targeting activity.

To assess vMAP function in apoptosis, polyclonal NIH3T3/puro and NIH3T3/vMAP stable cell lines were established. The expression of vMAP was confirmed by immunoblotting with anti-vMAP serum as shown in Figure 2D. These cells were treated with various apoptotic agents and stresses (staurosporine [ST], TNF-α, vesicular stomatitis virus infection, and nocodazole) to induce apoptosis, stained with propidium iodide (PI), and then analyzed by flow cytometry. vMAP expression significantly reduced the accumulation of sub-G1 cells that are considered to be apoptotic (Figure 2D and 2E). These results demonstrate that γHV-68 vMAP has robust anti-apoptotic activity toward various apoptotic agents.

### vMAP Interaction with Bcl-2 Family Proteins

**Figure 3:**
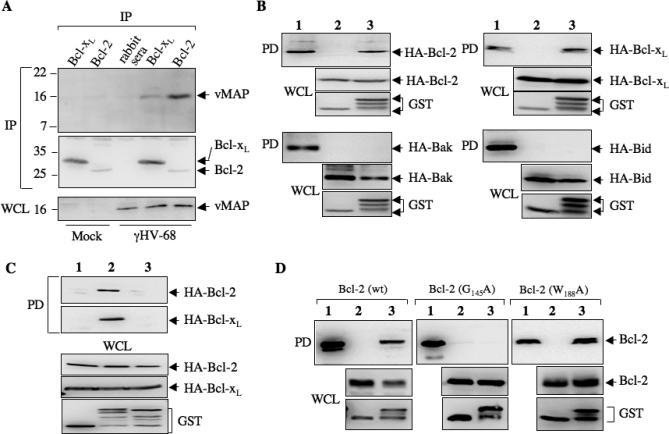
vMAP Interacts with Bcl-2/Bcl-x_L_ (A) vMAP interacts with Bcl-2/Bcl-x_L_ in γHV-68-infected cells. At 16 h post-infection, γHV-68-infected (MOI = 1) or mock-infected NIH3T3 cells were harvested and post-centrifuge supernatants were subjected to immunoprecipitation (IP) with rabbit antibodies to Bcl-2 or Bcl-x_L_, followed by immunoblotting with anti-vMAP serum. A normal rabbit serum was included as a negative control. Protein precipitates were analyzed by immunoblotting with antibodies to Bcl-2 and Bcl-x_L_ (middle panel). Whole cell lysates (WCL) of mock-infected or γHV-68-infected NIH3T3 cells were immunoblotted with anti-vMAP serum (bottom panel). (B) The N-terminal sequence of vMAP interacts with Bcl-2/Bcl-x_L_ but not with Bak and Bid. At 48 h post-transfection with a plasmid expressing HA-Bcl-2, HA-Bcl-x_L_, HA-Bak, or HA-Bid, and a plasmid expressing GST (lane 2) or vMAP(1–50)-GST (lane 3), cell lysates of 293T were used for GST pull-down (PD) assay, followed by immunoblotting with anti-HA (top panel of each set). WCLs were analyzed by immunoblotting with antibodies to HA epitope (Bcl-2 family proteins, middle panels) and GST (bottom panels) for GST or vMAP(1–50)-GST expression. Lane 1 is 2% input of lysates of cells expressing GST and Bcl-2 family proteins. For all four sets of PDs (top panel), lane 1 shows the equivalent amount of Bcl-2 family proteins compared to those shown in the middle panel. (C) The first 20 amino acids of vMAP are essential for its interaction with Bcl-2/Bcl-x_L_. 293T cells were transfected with a plasmid expressing HA-Bcl-2, HA-Bcl-x_L_ together with a plasmid expressing GST (lane 1), vMAP-GST (lane 2), or vMAPΔ20-GST (lane 3). Mammalian GST pull-down was carried out as described in (B). The precipitates were analyzed by immunoblotting with anti-HA to detect Bcl-2 and Bcl-x_L_ (top two panels). WCLs were analyzed by immunoblotting with anti-HA and anti-GST antibodies to demonstrate the expression of Bcl-2/Bcl-x_L_ and GST fusion, respectively. (D) The Bcl-2 BH2 domain is not involved in vMAP binding. At 48 h post-transfection with a plasmid expressing HA-Bcl-2, Bcl-2 G_145A_, or Bcl-2 W_188A_ together with a plasmid expressing GST (lane 2) or vMAP(1–50)-GST (lane 3), 293T cell lysates were used for GST pull-down, followed by immunoblotting with Bcl-2 antibody (top panel of each set). WCLs were analyzed by immunoblotting with antibodies to Bcl-2 (middle panel) and GST (bottom panel). Lane 1 indicates 2% input of lysates of cells expressing vMAP-GST and Bcl-2/Bcl-x_L_.

To investigate the molecular action of vMAP, we tested whether vMAP interacts with cellular apoptotic or anti-apoptotic proteins of the Bcl-2 family. Co-immunoprecipitation analyses showed that vMAP interacted with cellular Bcl-2 in transiently vMAP-expressing 293T cells and in γHV-68-infected NIH3T3 cells (Figures 3A and S3A). In addition, vMAP interaction with cellular Bcl-x_L_ was readily detected in γHV-68-infected NIH3T3 cells (Figure 3A). Despite the equivalent expression of three different species of vMAP in 293T cells, the 16-kDa vMAP predominantly interacted with Bcl-2, suggesting that the N-terminal sequence of vMAP is likely required for Bcl-2 interaction (Figure S3A). To further define the interaction between vMAP and Bcl-2 family proteins, a mammalian GST fusion protein containing vMAP (1–50) was coexpressed in 293T cells along with HA-tagged Bcl-2, Bcl-x_L_, Bax, Bak, Bid, or Bad. vMAP(1–50)-GST efficiently interacted with Bcl-2 and Bcl-x_L_ but not with Bax, Bak, Bad, or Bid (Figure 3B and unpublished data). However, vMAPΔ20-GST fusion lacking the N-terminal 20 amino acids completely lost its ability to bind to Bcl-2 and Bcl-x_L_ (Figure 3C). Interestingly, the N-terminal sequence containing the first 20 amino acids of vMAP is predicted to adopt an amphipathic helical structure and share limited similarity with the BH3 peptide of Bad (unpublished data). However, deletion mutation analysis indicated that unlike the BH3 peptide binding that requires the BH1, BH2, and BH3 domains of Bcl-2, the vMAP binding required the Bcl-2 85–186-aa region containing the BH1 and BH3 domains only in living cells (Figure S3B). This was further supported by the results that Bcl-2 G_145A_ mutation in the BH1 domain abolished vMAP binding, whereas Bcl-2 W_188A_ mutation in the BH2 domain did not affect vMAP binding (Figure 3D). By contrast, both mutations of Bcl-2 completely abrogated its Bid-binding activity under the same conditions (Figure S3C). This suggests that Bcl-2 binding to vMAP is different from its binding to BH3-only proteins.

### vMAP Interaction Facilitates Bcl-2 Mitochondrial Localization

**Figure 4:**
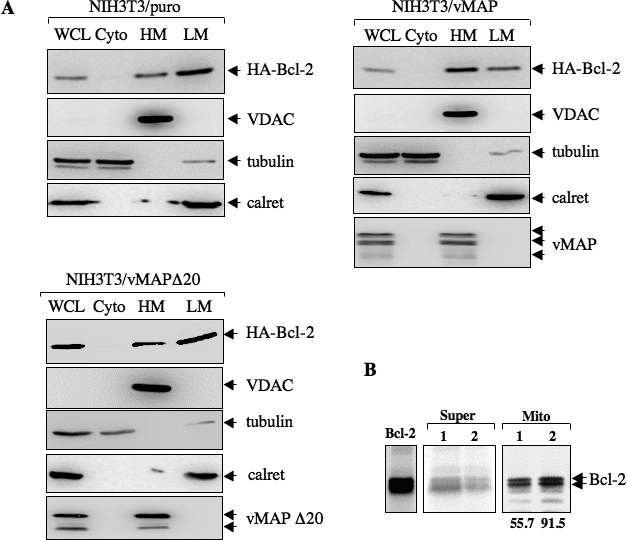
vMAP Enhances the Mitochondrial Localization of Bcl-2 (A) Bcl-2 intracellular distribution by subcellular fractionation. NIH3T3/puro, NIH3T3/vMAP, and NIH3T3/vMAPΔ20 cells were transfected with HA-Bcl-2 expression vector. Twenty μg of WCL, cytosolic (Cyto), heavy membrane (HM), and light membrane (LM) fractions were analyzed by immunoblotting with antibodies to HA epitope (HA-Bcl2, top panel), VDAC, tubulin, calreticulin (calret), or vMAP. (B) In vitro Bcl-2 mitochondrial association assay. The mitochondrion-enriched HM fractions of NIH3T3/puro (lane 1) or NIH3T3/vMAP cells (lane 2) were mixed with in vitro translated ^35^S-labeled Bcl-2 at 30 °C for 2 h, centrifuged at 13,000 rpm for 15 min to separate soluble (Super) and insoluble (Mito), followed by autoradiograph. The left panel indicates Bcl-2 translated in rabbit reticulocyte lysates. The numbers below autography indicate the levels (intensity of the Bcl-2 doublet) of mitochondrion-associated Bcl-2 determined by Phosphor Imager analysis.

Bcl-2 family members are found in the cytoplasm, the endoplasmic reticulum, and the nuclear membrane where they act as sensors of cellular damage or stress. Upon stress, members of Bcl-2 family proteins relocate to the mitochondrial surface where they exert their activity [6,18,19]. Thus, mitochondrial recruitment of Bcl-2 is considered an important step during the pro- or anti-apoptotic decision. To examine whether vMAP interaction affected Bcl-2 intracellular localization, the distribution of Bcl-2 was examined by subcellular fractionation. Whole-cell lysates were subjected to sequential centrifugation to obtain light membrane (LM) fraction containing microsomes derived from the endoplasmic reticulum or the trans Golgi network, HM fraction enriched with mitochondria, and cytosolic fraction. Densitometry quantification of immunoblotting revealed that approximately 70% and 30% of Bcl-2 was present in LM and HM of NIH3T3/puro cells, respectively, whereas 30% and 70% of Bcl-2 was present in the LM and HM of NIH3T3/vMAP cells, respectively (Figure 4A). vMAPΔ20 mutant that failed to interact with Bcl-2 showed only a little effect on Bcl-2 localization compared with wt vMAP (Figure 4A). It should be noted that despite the 20-aa deletion at the N-terminus, vMAPΔ20 mutant was still present primarily in the mitochondrion-enriched HM, as shown in Figures 4A and S2D, suggesting that vMAP may contain at least two independent motifs for its mitochondrial localization. To further test if vMAP recruited Bcl-2 into the mitochondrion in vitro, HM factions were used for mitochondrial association assay with [^35^S]-labeled Bcl-2 translated in rabbit reticulocyte lysates. Bcl-2 mitochondrial association activity increased approximately 2-fold in the HM fractions of NIH3T3/vMAP cells compared with those of NIH3T3/puro cells (Figure 4B). These results collectively indicate that vMAP actively recruits Bcl-2 to mitochondria, and that this vMAP activity requires the specific interaction with Bcl-2.

### vMAP Potentiates Bcl-2/Bcl-x_L_ to Neutralize BH3-Only Molecules

**Figure 5:**
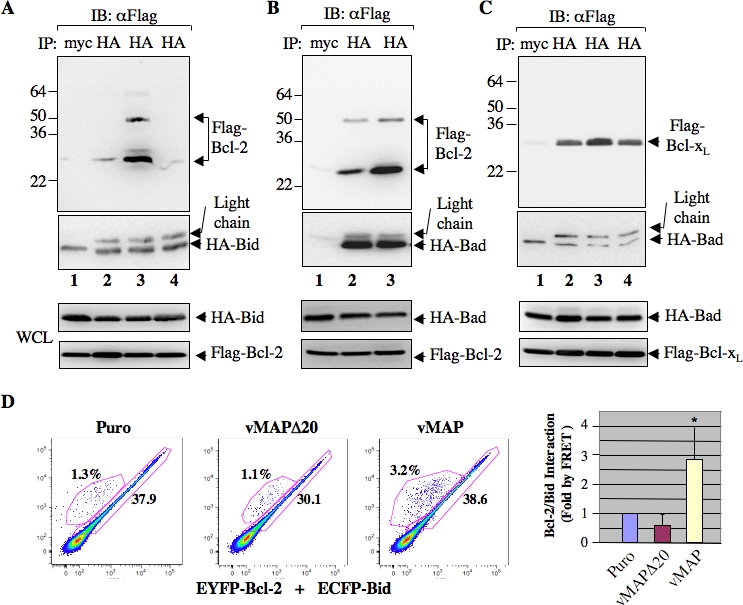
vMAP Enhances the Interaction between Bcl-2/Bcl-x_L_ and BH3-Only Proteins (A–C) NIH3T3/puro cells (lane 2), NIH3T3/vMAP cells (lanes 1 and 3), and NIH3T3/vMAPΔ20 cells (lane 4) were transfected with plasmids expressing HA-Bid and Flag-Bcl-2 (A), HA-Bad and Flag-Bcl-2 (B), or HA-Bad and Flag-Bcl-x_L_ (C). WCLs were used for immunoprecipitation with a mouse monoclonal anti-HA antibody, followed by immunoblotting with HRP-conjugated anti-Flag antibody to detect Flag-Bcl-2 and Flag-Bcl-x_L_ (top panel) or with anti-HA antibody to detect the precipitated HA-Bid and HA-Bad (second panels). WCLs were used to detect HA-Bid, HA-Bad, Flag-Bcl-2, and Flag-Bcl-x_L_ expression (bottom two panels) using antibodies to the HA epitope or Flag epitope. Lane 1 indicates anti-Myc immunoprecipitation of lysates from NIH3T3/vMAP cells as a negative control. The light chains of the Myc and HA antibodies migrate differently on SDS-PAGE. (D) Fluorescence resonance energy transfer (FRET) assay. At 36 h post-transfection with pEYFP-Bcl-2 and pECFP-Bid, NIH3T3/puro cells (Puro, panel 1), NIH3T3/vMAPΔ20 cells (vMAPΔ20, panel 2), and NIH3T3/vMAP cells (vMAP, panel 3) were harvested and subjected to flow cytometry analysis. Numbers in the FRET diagrams indicate the percentage of the transfected cell population without protein interaction (lower right) and the transfected cell population with protein interaction (upper left). The right panel shows the data in the graph representing an average from two independent experiments, and error bars indicate standard deviation with (*) *p* < 0.05 relative to control (Puro) as calculated by Student's *t*-test.

The ability to associate with BH3-only molecules correlates with the anti-apoptotic activity of Bcl-2 and Bcl-x_L_ [20,21]. Because vMAP facilitated the mitochondrial recruitment of Bcl-2, we tested whether vMAP expression affected the ability of Bcl-2 and Bcl-x_L_ to associate with BH3-only molecules. NIH3T3/puro and NIH3T3/vMAP cells were transfected with Flag-tagged Bcl-2 and HA-tagged Bad or Bid. At 36 h post-transfection, cell lysates were used for immunoprecipitation with anti-HA, followed by immunoblotting with anti-Flag. Surprisingly, the interaction of Bcl-2 with Bad or Bid was considerably higher in NIH3T3/vMAP cells than in NIH3T3/puro cells (Figure 5A and 5B). vMAP expression also had a similar effect on the Bcl-x_L_-Bad interaction (Figure 5C). In contrast, vMAP expression affected neither Bcl-2/Bcl-x_L_ interaction with Bax/Bak, nor Bcl-x_L_ dimerization under the same conditions (Figure S3D, S3E, and unpublished data). vMAPΔ20, which failed to interact with Bcl-2 and Bcl-x_L_ but still localized at mitochondria, had no effect on their interactions with Bid or Bad (Figure 5A and 5C, lane 4).

To further demonstrate the effect of vMAP on Bcl-2 interaction with BH3-only molecules in living cells, a flow cytometry–based fluorescence resonance energy transfer (FRET) assay was used to quantify the interaction between Bcl-2 and Bid. NIH3T3/puro, NIH3T3/vMAP, and NIH3T3/vMAPΔ20 cells were transfected with EYFP-Bcl-2 and ECFP-Bid expression vectors. At 36 h post-transfection, these cells were subjected to a FRET assay. The Bcl-2–Bid interaction increased approximately 2- to 3-fold in NIH3T3/vMAP cells as compared with NIH3T3/puro cells, whereas no significant difference was detected between NIH3T3/puro and NIH3T3/vMAPΔ20 cells (Figure 5D). Furthermore, consistent with immunoprecipitation results (Figure S3D and S3E), vMAP did not significantly alter the Bcl-2–Bax interaction in the FRET assay (Figure S3F). These results demonstrate that vMAP specifically facilitates the interactions of Bcl-2/Bcl-x_L_ with Bid/Bad.

### vMAP Inhibits the Activation and Mitochondrial Translocation of Bax

**Figure 6:**
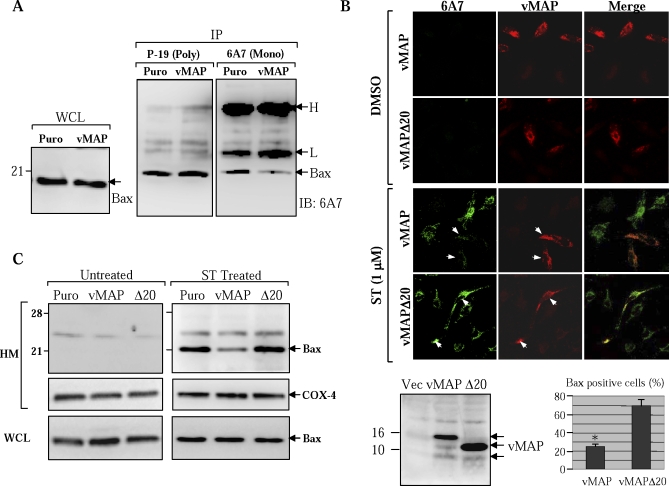
vMAP Inhibits the Mitochondrial Translocation and Activation of Bax (A) vMAP inhibits the apoptotic conformational change of Bax. NIH3T3/puro (Puro) and NIH3T3/vMAP (vMAP) cells were treated with ST (1 μM) for 4 h, harvested, and lysed in 1% CHAPS buffer. Pre-cleared cell lysates were split into two fractions, and each was used for immunoprecipitation with the mouse 6A7 monoclonal antibody or the rabbit P-19 polyclonal antibody, followed by immunoblotting with 6A7. WCLs were analyzed by immunoblotting with 6A7 antibody. Of note, 6A7 antibody reacts only with the activated Bax in immunoprecipitation and immunofluorescence assays, but with pan-bax in the immunoblotting assay. H and L indicate the heavy and light chains of immunoglobulin, respectively. WCL, whole cell lysate. (B) vMAP inhibits Bax activation by immunofluorescence assay. At 16 h post-transfection with a plasmid expressing vMAP or vMAPΔ20, HeLa cells were treated with DMSO or ST (1 μM) for 4 h, fixed, and stained with rabbit anti-vMAP serum (red) and the mouse 6A7 monoclonal anti-Bax antibody (green). A single representative optical section is presented. Arrows indicate cells expressing vMAP or vMAPΔ20. The bottom right graph represents data collected from over 200 transfected cells. Error bars indicate standard deviation with (*) *p* = 0.01 relative to vMAPΔ20 as calculated by Student's *t*-test. The bottom left panel shows the expression of vMAP and vMAPΔ20 (Δ20). (C) vMAP inhibits Bax translocation by fractionation. NIH3T3/puro cells (Puro), NIH3T3/vMAP cells (vMAP), or NIH3T3/vMAPΔ20 cells (Δ20) were untreated or treated with ST (1 μM) for 4 h, and the mitochondrion-enriched HM fractions (20 μg) were resolved by SDS-PAGE and analyzed by immunoblotting with antibodies to detect endogenous Bax and COX4. WCLs were analyzed by immunoblotting with anti-Bax antibody (bottom panel).

BH3-only pro-apoptotic proteins transduce death signals from the cell surface or intracellular apoptotic pathways by inducing a conformational change in Bax. Subsequently, Bax translocates to mitochondria and oligomerizes within the outer membrane, which ultimately leads to membrane permeabilization and release of pro-apoptotic factors from the intermembrane space [5,22]. To test if vMAP expression affected Bax activation, mouse monoclonal antibody 6A7 that specifically recognizes an epitope in the pro-apoptotic Bax conformer was used to assess the level of Bax activation [23]. NIH3T3/puro and NIH3T3/vMAP cells were treated with ST (1 μM) for 4 h, lysed with 1% CHAPS buffer, and subjected to immunoprecipitation with the 6A7 monoclonal antibody or the P-19 rabbit polyclonal antibody that reacts with total Bax. NIH3T3/vMAP cells reproducibly showed lower levels of the pro-apoptotic Bax conformer (Figure 6A). This difference was not due to a reduced level of Bax expression, as immunoprecipitation and immunoblotting with the P-19 antibody showed the equivalent amounts of Bax in both cells (Figure 6A). Confocal immunofluorescence microscopy also showed that vMAP expression substantially suppressed the ST-induced Bax activation in HeLa cells (Figure 6B). In contrast, vMAPΔ20, which failed to interact with Bcl-2/Bcl-x_L_, did not affect Bax activation (Figure 6B). Quantification of 6A7 Bax antibody–positive cells showed that over 70% of vMAPΔ20-expressing cells were positive to the 6A7 Bax conformer antibody at 4 h after ST treatment, whereas only 25% of vMAP-expressing cells were positive (Figure 6B).

Subcellular fractionation was further used to examine the mitochondrial translocation of Bax. NIH3T3/puro, NIH3T3/vMAP, and NIH3T3/vMAPΔ20 cells were treated with ST (1 μM) for 4 h, and equivalent amounts of HM were used for immunoblotting. Upon ST treatment, endogenous Bax efficiently translocated into mitochondria in NIH3T3/puro and NIH3T3/vMAPΔ20 cells, whereas a significant reduction of Bax mitochondrial translocation was detected in NIH3T3/vMAP cells (Figure 6C). Equivalent amounts of Bax expression were detected in all three cells (Figure 6C). Collectively, these data indicate that vMAP expression significantly suppresses Bax activation as well as its mitochondrial translocation.

### The Central and C-Terminal Hydrophobic Regions of vMAP Interact with VDAC1

**Figure 7:**
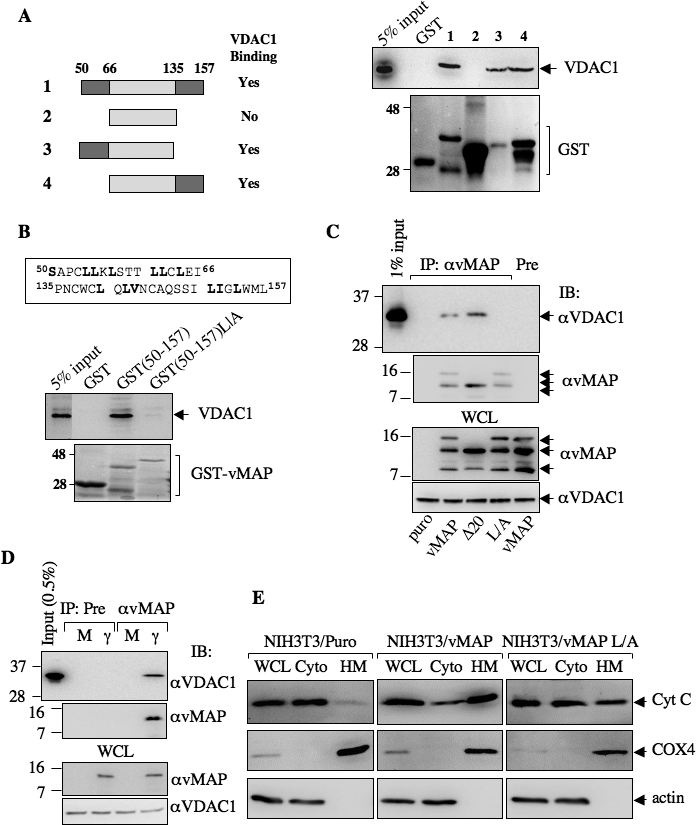
vMAP Interaction with VDAC1 Inhibits Cytochrome c Release (A) vMAP contains two VDAC1-interacting regions (dark grey boxes). The left panel shows the schematic diagram of vMAP interaction with VDAC1. (Right panel) GST fusions containing various vMAP sequences were expressed and purified from E. coli. 293T cells were lysed in CHAPS buffer and subjected to in vitro GST pull-down, followed by immunoblotting with anti-VDAC antibody. The bottom panel shows the Coomassie blue staining of GST fusion proteins. Lanes 1–4 correspond to the GST fusions shown by the left diagram. (B) The LLxL repeat sequences of vMAP are required for VDAC1 interaction. (Top box) The boxed sequences are the two LLxL repeats of vMAP. GST or GST fusion proteins were used to bind to VDAC1 from 293T cell lysates in CHAPS buffer as described in (A). Protein precipitates were analyzed by immunoblotting with anti-VDAC antibody (top blot) and Coomassie blue staining of GST fusion proteins (bottom blot). (C) vMAP interacts with VDAC1 in stable cell lines. NIH3T3 cells stably expressing vector, vMAP, or its mutants were used for immunoprecipitation with anti-vMAP serum or pre-immune (Pre) serum, followed by immunoblotting with anti-VDAC antibody (top panel) or anti-vMAP serum (middle panel). WCLs were analyzed by immunoblotting with anti-vMAP serum and anti-VDAC antibody (bottom two panels). (D) vMAP interacts with VDAC1 in γHV-68-infected cells. WCLs of mock (M)- or γHV-68 (γ)-infected (MOI = 1) cells were precipitated with anti-vMAP or preimmune serum, followed by immunoblotting with anti-VDAC antibody (top panel) or anti-vMAP serum (middle panel). WCLs were analyzed by immunoblotting with anti-vMAP serum and anti-VDAC antibody (bottom two panels). (E) vMAP inhibits cytochrome c release upon ST treatment. NIH3T3/puro, NIH3T3/vMAP, or NIH3T3/vMAP L/A cells were treated with ST (1 μM) for 4 h and WCLs were sequentially centrifuged to obtain cytosolic (Cyto) and mitochondrion-enriched heavy membrane (HM) fractions. Polypeptides (20 μg) from each fraction were analyzed by immunoblotting with antibodies to cytochrome c (Cyt C), COX4, and actin.

While the N-terminal 50 residues of vMAP were sufficient for interacting with Bcl-2 and this interaction exhibited a pleiotropic effect on Bcl-2 family proteins, the loss of Bcl-2 interaction did not completely impede vMAP-mediated anti-apoptosis (see below). This suggests that vMAP might have additional cellular targets to achieve anti-apoptotic activity. To test this idea, we used the yeast two-hybrid screen with vMAP 50–157-aa region as bait to search for vMAP-interacting cellular protein(s). This study identified cellular VDAC1 as a vMAP-interacting protein. An in vitro GST pull-down experiment showed that vMAP specifically bound to cellular VDAC1 (Figure 7A, right panel, lane 1).

vMAP contains two leucine-rich (LLxL, LIxL, and LxLV) hydrophobic regions consisting of residues 50–66 and 135–157 (Figure 7A, dark grey box). To test whether these hydrophobic regions mediated the interaction with VDAC1, bacterial GST-vMAP(50–157), GST-vMAP(66–135), GST-vMAP(50–135), and GST-vMAP(66–157) fusion proteins were used for in vitro GST pull-down assays, followed by immunoblotting with antibody to VDAC1. GST fusions containing either hydrophobic region 50–66 or 135–157 effectively interacted with endogenous VDAC1. GST alone or GST-vMAP(66–135) did not interact with VDAC1 under the same conditions (Figure 7A). Furthermore, the vMAP L/A mutant carrying the replacement of the leucine and isoleucine residues in both hydrophobic regions with alanines no longer interacted with VDAC1 (Figure 7B). NIH3T3 cells stably expressing wt vMAP, vMAPΔ20, or vMAP L/A at equivalent levels (Figure S2E) were tested for vMAP and VDAC1 interaction. Both vMAP wt and vMAPΔ20 efficiently interacted with VDAC1, whereas vMAP L/A mutant did not interact with VDAC1, indicating that the leucine-rich motifs within the hydrophobic regions of vMAP are required for its interaction with VDAC1 (Figure 7C). Finally, vMAP interaction with VDAC1 was readily detected in γHV-68-infected NIH3T3 (Figure 7D).

Cellular VDAC1 is located at the mitochondrial outer membrane and has a role in the release of pro-apoptotic factors such as cytochrome c upon apoptotic stress [24]. To test whether the vMAP–VDAC1 interaction affected cytochrome c release, NIH3T3/puro and NIH3T3/vMAP cells were treated with ST for 4 h and subjected to intracellular fractionation, followed by immunoblotting with antibodies to cytochrome c and COX4. This showed the significant release of mitochondrial cytochrome c to the cytosol in NIH3T3/puro cells, whereas only minimal cytochrome c leaked from mitochondria in NIH3T3/vMAP cells (Figure 7E). Finally, NIH3T3/vMAP L/A cells displayed considerable release of mitochondrial cytochrome c upon ST treatment; however, the extent of release was relatively lower in NIH3T3/vMAP L/A cells than in NIH3T3/puro cells (Figure 7E). Taken together, these results indicate that vMAP interacts with mitochondrial outer membrane VDAC1 and that this interaction robustly inhibits cytochrome c release.

### Effect of Bcl-2 and VDAC1 on vMAP-Mediated Anti-Apoptotic Activity

**Figure 8:**
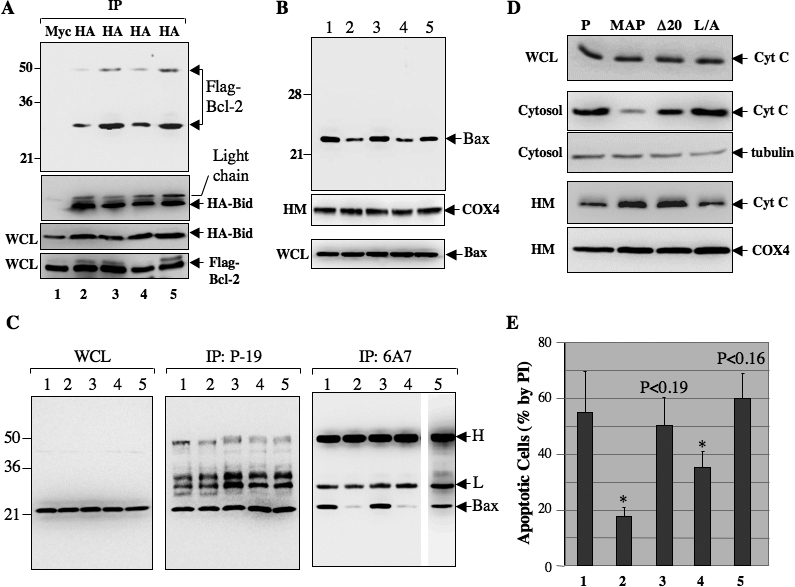
vMAP Interacts with Bcl-2 and VDAC1 in a Genetically Separable Manner (A) vMAP interaction with Bcl-2, but not with VDAC1, enhances Bcl-2 binding to Bid. NIH3T3 stable cells expressing puro (lane 2), vMAP (lanes 1 and 3), vMAPΔ20 (lane 4), or vMAP L/A (lane 5) were transfected with plasmids containing Flag-Bcl-2 and HA-Bid. At 48 h post-transfection, cells were harvested and WCLs were used for immunoprecipitation with anti-HA (Bid), followed by immunoblotting with HRP-conjugated anti-Flag (Bcl-2, top panel) or anti-HA antibody (middle panel). WCLs were analyzed by immunoblotting with anti-HA (Bid) and anti-Flag (Bcl-2) antibodies (bottom two panels). Anti-Myc antibody in lane 1 was included as a negative control. (B) vMAP-Bcl-2 interaction but not vMAP-VDAC1 interaction is required to inhibit Bax mitochondrial translocation. NIH3T3 stable cells were treated with ST (1 μM) for 4 h, and mitochondrion-enriched HMs (20 μg) were analyzed by immunoblotting with anti-Bax or anti-VDAC antibody (top two panels). WCLs were used for immunoblotting with anti-Bax (bottom panel). Lane 1, NIH3T3/puro; lane 2, NIH3T3/vMAP; lane 3, NIH3T3/vMAPΔ20; lane 4, NIH3T3/vMAP L/A; lane 5, NIH3T3/vMAPΔ20&L/A. (C) vMAP-Bcl-2 interaction but not vMAP-VDAC1 interaction is required to inhibit the mitochondrial translocation of Bax. NIH3T3 cells expressing wt vMAP or mutants thereof were stimulated with ST (1 μM) for 4 h. WCLs were precipitated with anti-Bax P-19 polyclonal antibody or 6A7 monoclonal antibody, followed by immunoblotting with 6A7 monoclonal antibody as described in Figure 6A. The content of the lanes is same as described in (B). H and L indicate the heavy and light chains of immunoglobulin, respectively. (D) vMAP interactions with Bcl-2 and VDAC1 are required to efficiently inhibit cytochrome c release. NIH3T3 cells containing vector (P), vMAP, vMAPΔ20 (Δ20), or vMAP L/A (L/A) were treated with ST (1 μM) for 4 h. Mitochondrion-enriched HM and cytosolic fractions (Cytosol) were obtained by centrifugation as described in Materials and Methods. Each fraction (20 μg) was resolved by SDS-PAGE and analyzed by immunoblotting with antibodies to cytochrome c (Cyt C), COX4, and tubulin (cytosolic fraction only). (E) Roles of vMAP interactions with Bcl-2 and VDAC1 in anti-apoptosis. NIH3T3 cells were treated with ST (1 μM) for 16 h and subsequently stained with PI for flow cytometry. Data represent three independent experiments. Error bars indicate standard deviation with (*) *p* < 0.05 relative to control (NIH3T3/puro cells, 1) as calculated by Student's *t*-test. The content of the lanes is same as described in (B).

The biological significance of the vMAP interactions with Bcl-2/Bcl-x_L_ and VDAC1 in anti-apoptosis was tested by examining the effect of wt vMAP and its mutants (Δ20, L/A, Δ20&L/A) on Bcl-2–Bid interaction, Bax translocation and activation, cytochrome c release, and apoptosis (PI staining). While vMAPΔ20 had no effect on the Bcl-2–Bid interaction and Bax translocation/activation, the vMAP L/A mutant potentiated the Bcl-2–Bid interaction and inhibited Bax translocation/activation as efficiently as wt vMAP (Figure 8A–8C). Functionally, vMAP activity to inhibit cytochrome c release was detectably impaired by both mutations (Δ20 and L/A), consistent with the finding that Bax permeabilizes the mitochondrial outer membrane to release cytochrome c (Figure 8D). These data indicate that vMAP targets two mitochondrial apoptosis checkpoint proteins, Bcl-2/Bcl-x_L_ and VDAC1, in a genetically separable manner.

To further investigate the significance of vMAP interactions with Bcl-2 and VDAC1 in the inhibition of apoptosis, NIH3T3 cells expressing wt vMAP or its mutants were treated with ST for 16 h. ST treatment induced extensive apoptosis in NIH3T3/puro cells, whereas wt vMAP efficiently blocked ST-induced apoptosis (Figure 8E). In contrast, L/A and Δ20 mutations significantly impaired the anti-apoptotic activity of vMAP under the same conditions (Figure 8E). Finally, the Δ20&L/A double mutations completely abrogated the ability of vMAP to inhibit ST-induced apoptosis (Figure 8E). These results indicate that the Bcl-2 interaction displays a more pronounced role in the vMAP-mediated anti-apoptosis than the VDAC1 interaction, and both interactions lead to the comprehensive inhibition of the mitochondrion-mediated apoptosis.

### vMAP Is Required for Efficient Lytic Replication of γHV-68 in Normal Fibroblasts but Not in Bax/Bak-Deficient Fibroblasts

**Figure 9:**
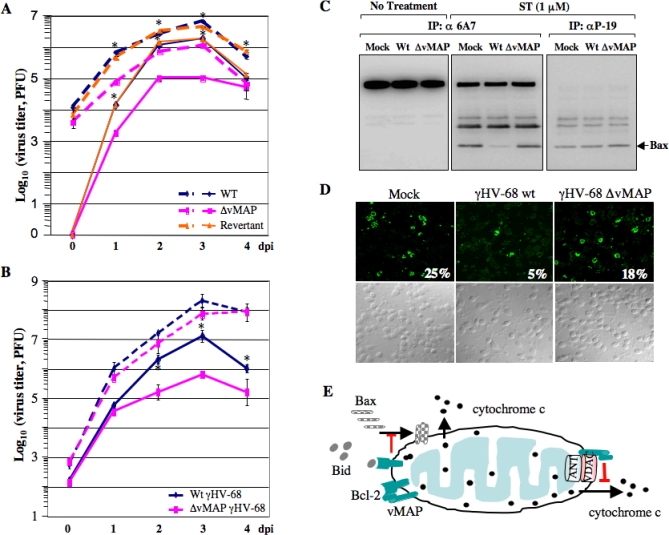
vMAP Anti-Apoptotic Activity Is Required for the Efficient Replication of γHV-68 (A) vMAP is required for the efficient replication of γHV-68 in NIH3T3 cells. NIH3T3 cells were infected with γHV-68 wt, γHV-68 ΔvMAP, or γHV-68 Rev at MOI = 0.01 (solid line) or MOI = 5 (dashed line). Culture (cells and supernatants) was harvested at various time points post-infection. Plaque assay was performed with BHK21 cells. Data represent duplicate experiments and error bars indicate standard deviation with (*) *p* < 0.07 relative to γHV-68 ΔvMAP as calculated by Student's *t*-test. dpi, days post infection. The *p*-value can be applied for both γHV-68 wt and γHV-68 Rev. (B) vMAP is dispensable for γHV-68 replication in Bax^−/−^Bak^−/−^ (DKO) MEF. wt or Bax^−/−^Bak^−/−^ (DKO) MEF cells were infected with γHV-68 wt or γHV-68 ΔvMAP at MOI = 1. Culture (cells and supernatants) was harvested at various time points post infection. Solid lines and dashed lines indicate growth kinetics of γHV-68 in wt MEFs and DKO MEFs, respectively. Data represent the results of two independent measurements and error bars indicate standard deviation with (*) *p* < 0.06 relative to wt MEF as calculated by Student's *t*-test. (C and D) vMAP expression inhibits Bax activation during γHV-68 replication. NIH3T12 cells were mock-infected (Mock), infected with γHV-68 wt (Wt) or γHV-68 ΔvMAP (ΔvMAP) at MOI = 2 for 12 h, and then untreated or treated with ST at 1 μM for 4 h. Bax activation was examined by immunoprecipitation (P-19 and 6A7) and immunoblotting (6A7) (C) or confocal microscopy with 6A7 monoclonal antibody (D) as described in Figure 5. Numbers in (D) indicate the percentages of 6A7 Bax conformer positive cells. (E) A hypothetical model of vMAP action in the inhibition of mitochondrion-mediated apoptosis. γHV-68 vMAP recruits Bcl-2 to the mitochondrion and enhances Bcl-2 interaction with BH3-only proteins, thereby blocking Bax translocation and activation. On the other hand, vMAP interactions with both Bcl-2 and VDAC1 lead to a comprehensive inhibition of cytochrome c release upon apoptotic stress. Red-colored lines indicate vMAP-mediated inhibition.

To investigate the effect of vMAP on γHV-68 lytic replication, the bacterial artificial chromosome system was used to generate recombinant γHV-68 ΔvMAP that contained the removal of first Met_1_ and second Met_21_ residues and the insertion of a stop codon without affecting ORF57 coding sequences (for details of nucleotide changes, please see Materials and Methods). γHV-68 ΔvMAP Kan^R^ Bac was initially constructed and subsequently used to generate γHV-68 ΔvMAP and the revertant virus, called γHV-68 Rev, which contained wt vMAP sequence (Figure S4A). vMAP protein was readily detected in wt and revertant γHV-68-infected cells but not in γHV-68 ΔvMAP-infected cells (Figure S4B). To test if vMAP played a role in virus lytic replication, wt γHV-68, γHV-68 ΔvMAP, and γHV-68 Rev were used to infect NIH3T3, wt MEF, and Bax^−/−^Bak^−/−^ DKO MEF cells and their replication kinetics were determined by plaque assay. γHV-68 ΔvMAP replicated at levels that were 10- to 30-fold lower in NIH3T3 and wt MEF cells throughout replication cycle than they were in wt γHV-68 and γHV-68 Rev (Figure 9A and 9B). However, this reduced replication capacity of γHV-68 ΔvMAP was considerably diminished in Bax^−/−^Bak^−/−^ DKO MEFs: γHV-68 ΔvMAP replicated at similar kinetic and slightly reduced peak titer compared to wt γHV-68 (Figure 9B).

To further define the role of vMAP in mitochondrial cell death during viral replication, mouse fibroblast cells were infected with wt γHV-68 or γHV-68 ΔvMAP, and Bax activation was then examined by immunoprecipitation and confocal microscopy with 6A7 Bax conformer antibody. After treatment with ST (1 μM, 4 h), wt γHV-68-infected cells showed the greatly reduced level of activated Bax compared to mock-infected or γHV-68 ΔvMAP-infected cells (Figure 9C and 9D). These results demonstrate that the vMAP gene is required for efficient γHV-68 lytic replication in normal fibroblast cells, but not in mitochondrial apoptosis-deficient fibroblast cells. This indicates that vMAP may serve a vital role in γHV-68 lytic replication by inhibiting the premature mitochondrial apoptotic death of host cells during acute replication, allowing completion of viral replication cycle.

## Discussion

Here, we report the identification of a novel mitochondrial anti-apoptotic vMAP of γHV-68, of which its N-terminal MTS is sufficient for the mitochondrial localization. vMAP interacts with Bcl-2 and increases Bcl-2 mitochondrial localization, leading to the neutralization of BH3-only pro-apoptotic molecules. Additionally, vMAP binds to the mitochondrial VDAC1 through its internal and C-terminal hydrophobic sequences, thereby inhibiting cytochrome c release upon apoptotic stresses. Taken together, these data indicate that vMAP engages cellular Bcl-2 and VDAC apoptosis checkpoint proteins to comprehensively inhibit the mitochondrion-mediated intracellular innate immunity, which allows completion of efficient viral lytic replication (Figure 9E).

Previous functional studies have classified BH3-only proteins as either death agonists such as Bid and Bim or survival antagonists like Bad [22,25]. Recently, this has been formally proposed as the “hierarchy model”, which postulates that the survival antagonist mainly promotes apoptosis by neutralizing anti-apoptotic Bcl-2 members, the death agonist induces a conformational change, oligomerization, and activation of Bax/Bak through a “hit-and-run” mechanism, thereby amplifying apoptotic signaling with a limited amount of cleaved Bid or dephosphorylated Bim [26]. In normal cells, the death agonist is held in check by anti-apoptotic Bcl-2 family proteins. When apoptosis is triggered, the survival antagonist binds to anti-apoptotic Bcl-2 proteins that release the death agonist, which subsequently activates Bax or Bak. This model places the survival antagonist upstream of the death agonist. Thus, BH3-only proteins display a synergistic effect in activating Bax/Bak and inducing apoptosis [22,25]. Our data is in support of the hierarchy model regarding the action of BH3-only molecules. Conceivably, vMAP interaction may activate Bcl-2/Bcl-x_L_ to adopt their anti-apoptotic conformation, effectively neutralizing the pro-apoptotic BH3-only molecules through a direct interaction, which ultimately blocks Bax activation. It has been shown that cellular orphan nuclear receptor Nur77 has an activity to modulate Bcl-2 conformation and convert Bcl-2 into a pro-apoptotic molecule [27]. This suggests that vMAP may resemble Nur77 by altering cellular Bcl-2/Bcl-x_L_ conformation, but this activity transforms Bcl-2 into an anti-apoptotic conformation rather than a pro-apoptotic form. However, it should be noted that the vMAP exhibits no specificity in Bcl-2 interactions with the death agonist Bid or the survival antagonist Bad. It is possible that vMAP increases the pool of activated anti-apoptotic Bcl-2 in general, which is reflected by the elevated interaction with Bid as well as Bad. In contradiction to the hierarchy model, BH3-only molecules have recently been proposed to induce apoptosis primarily through neutralization of Bcl-2 anti-apoptotic proteins, but not through activation of Bax/Bak [28]. The reason for this seemingly discrepancy between these two models is not clear and may be derived from various different mutant proteins and functional tests that each study relied on [26,28]. Thus, further studies are required to resolve this issue regarding BH3-only molecules. Nevertheless, our data indicate that the vMAP facilitates the interactions of Bcl-2/Bcl-x_L_ with Bid/Bad BH3-only molecules, which neutralizes Bid/Bad pro-apoptotic activity, inhibits Bax activation, and thereby likely raises the threshold for cells to execute apoptosis.

In addition to Bax/Bak activation, the PTP complex represents an additional apoptotic checkpoint within the mitochondrial membrane. Along with accessory components, the PTP complex is mainly composed of ANT and VDAC that connect the mitochondrial outer membrane with its inner membrane at contact sites. Although ANT and VDAC may not be required for mitochondrion permeabilization, accumulating data indicate that they are implicated in releasing pro-apoptotic factors from the intermembrane space [7,24,29,30]. Cellular Bcl-2 family proteins and viral polypeptides differentially modulate the PTP complex through a direct interaction with ANT, VDAC, or their accessory components, and therefore regulate apoptosis [7,31–33]. Our present study adds vMAP to the expanding family of proteins that influence the permeability transition by virtue of protein–protein interactions. The two vMAP LLxL repeats independently mediate an interaction with VDAC1 that is required to efficiently inhibit cytochrome c release, an indication of the mitochondrial permeability transition.

We have defined two functional domains within vMAP: the N-terminal Bcl-2-binding domain and the central/C-terminal VDAC1 interaction domain. It is reasonable to speculate the potential presence of a ternary complex consisting of vMAP, Bcl-2, and VDAC1. Given that Bcl-2 physically and functionally interacts with VDAC1 [7], the introduction of vMAP to this complex may influence the Bcl-2/VDAC1 interaction if a ternary complex is present. However, we observed no detectable effect of vMAP on the interaction between Bcl-2 and VDAC1 (Figure S5). On the other hand, it is also possible that vMAP independently binds to cellular Bcl-2 or VDAC1. Consistent with this, the aforementioned two binding domains of vMAP are genetically separable in that mutations within each domain only affect its corresponding interaction, leaving the other interaction intact. Alternatively, the formation of a ternary complex may be dependent on the integrity of a lipid bilayer. Therefore, additional approaches other than traditional immunoprecipitation are required to assess vMAP interactions with Bcl-2 and VDAC1.

While both Bcl-2 and VDAC1 interactions are essential for vMAP-mediated inhibition of apoptosis, Bcl-2 binding seems to be more functionally important in vMAP-mediated inhibition of apoptosis than that of VDAC1. This is consistent with the findings that Bax and Bak are the essential players that open the mitochondrial gate to the cell death program [6,9]. In addition, Bcl-2 family proteins are also important in regulating cytochrome c release during apoptosis. Indeed, vMAPΔ20 that no longer bound to Bcl-2 partially lost its activity to inhibit cytochrome c release (Figure 8C). While vMAP L/A mutant that no longer bound to VDAC1 significantly failed to block cytochrome c release, a detectable amount of cytochrome c was still retained in the mitochondrion in these cells (Figure 7E). These data support the idea that vMAP interaction with Bcl-2 also plays a role in the inhibition of cytochrome c release. Taken together, these results indicate that vMAP interactions with both Bcl-2 and VDAC1 synergistically contribute to its inhibition on the mitochondrion-mediated apoptosis.

The N-terminal MTS of vMAP entirely overlaps with its Bcl-2 binding motif. This suggests that the N-terminal deletion mutation, vMAPΔ20, may ablate two functions of vMAP simultaneously: Bcl-2 binding and mitochondrial targeting. However, the results of cell fractionation and confocal microscopy showed that the majority of vMAPΔ20 still localized to the mitochondrion (Figures 4A and S2D). This indicates that vMAPΔ20 mutation ablates only the Bcl-2 binding activity without significantly affecting vMAP mitochondrial localization. This also suggests the presence of additional mitochondrial targeting sequence. Unfortunately, our GFP fusion strategy failed to identify additional linear sequence for its mitochondrial localization (unpublished data). This implies that, perhaps, a higher order of structure of vMAP may be required to function as an MTS as seen with VDAC [34]. The cleavable N-terminal MTS generally sends protein into the matrix or interior membrane of mitochondria, while the uncleavable N-terminal MTS targets protein into the outer membrane of mitochondria [35]. In fact, vMAP N-terminal MTS appeared to be not cleaved based on its molecular weight in SDS-PAGE (Figures 2A and 4A). Additionally, a mutation at the potential mitochondrial signal peptide cleavage site of vMAP did not affect its mitochondrial localization (unpublished data). These results collectively suggest that an alternative MTS exists in addition to the N-terminal MTS.

The expression profile and function activity of γHV-68 vMAP has unique features. First, vMAP is present within the second exon of the ORF57 transcript, which encodes an immediate early gene product that is essential for viral replication, a homolog of herpes simplex virus ICP27 [17,36]. This suggests that the vMAP gene has co-evolved with ORF57 and likely plays a critical role in γHV-68 replication. Second, the putative N-terminal amphipathic α-helical region of vMAP has both mitochondrial targeting and Bcl-2-binding activities. Of note, the vMAP contains an unidentified MTS, in addition to its N-terminal MTS, that potentially targets vMAP to the outer membrane of mitochondria. Finally, vMAP targets two mitochondrial proteins, Bcl-2 and VDAC1, to affect a comprehensive inhibition of the mitochondrion-dependent apoptosis. Interestingly, human cytomegalovirus (HCMV) vMIA uses a similar strategy to deregulate mitochondrion-dependent apoptosis: vMIA neutralizes and inhibits permeability transition pore activity through its direct interactions with Bax [32,37,38]. Intriguingly, vMIA is also encoded within the first exon of UL37 of HCMV, an immediate early gene transcript that is required for viral replication [39,40]. However, inactivating the vMIA expression by mutagenesis did not dramatically reduce HCMV lytic replication, similar to what we reported here for vMAP deletion for γHV-68 lytic replication in tissue culture [41]. Thus, it is surprising that these viruses have undergone convergent evolution, evolving independently to encode mitochondrial proteins with similar molecular mechanisms but without discernable sequence similarity.

## Materials and Methods

### Cells, virus, and plaque assays.

NIH3T3, NIH3T12, BHK21, COS-1, and 293T cells were grown in Dulbecco's modified Eagle's medium (DMEM) supplemented with 10% fetal bovine serum, 2 mM l-glutamine, 100 U/ml streptomycin and penicillin. BJAB and S11 cells were grown in RPMI 1640 supplemented with 10% fetal bovine serum, l-glutamine, and antibiotics. Fugene 6 (Roche), lipofectamine (Invitrogen), or calcium phosphate (Clontech) was used for transient expression of vMAP in COS-1, NIH3T3, and 293T cells. NIH3T3 stable cell lines were established using a standard protocol of puromycin selection at 2 μg/ml. wt MEFs or Bax^−/−^Bak^−/−^ DKO MEFs transformed with SV40 were generously provided by Stanley Korsmeyer (Dana-Farber Cancer Institute, Harvard Medical School) and grown in complete DMEM medium.

γHV-68 wt and GFP viruses were amplified in NIH3T12 cells. After three rounds of freeze-thawing, supernatants of virus-infected cells were used for de novo infection of BHK21 or NIH3T12 cells. Plaque assays were performed in BHK21 cells overlaid with 1% methylcellulose as described previously [42].

### γHV-68 recombinant virus.

γHV-68ΔK3-GFP was kindly provided by Dr. Philip Stevenson. To generate vMAP deletion recombinant virus, the bacterial artificial chromosome [43] containing the entire γHV-68 genome was used. Due to the fact that the vMAP coding sequence overlaps with ORF57, we generated γHV-68 ΔvMAP by changing the first and second initiation codons of vMAP to AAG and introducing two stop codons (T > A changes at nucleotide positions of 76016, 76049, 76076, and a G > A change at nucleotide position of 76079 based on Entrez accession number U97553). These changes did not affect the ORF57 coding sequence. Initially, the vMAP coding sequence was replaced with a Kan^R^/*Lac*Z cassette (γHV-68 Bac ΔvMAP Kan^R^) by allelic exchange [42] (Figure S4A). Since ORF57 was required for γHV-68 replication, vMAP sequences containing various mutations were amplified by PCR and electroporated into BHK21 cells together with γHV-68 Bac ΔvMAP Kan^R^ DNA to allow homologous recombination. Viruses obtained from BHK21 cells, γHV-68 ΔvMAP or γHV-68 Rev, were plaque purified and further amplified in BHK21 cells (Figure S4A). Virion DNAs were used for PCR amplification to clone ORF57 and vMAP DNA fragments, followed by DNA sequence to confirm the presence of designed changes and the absence of aberrant changes. Virion DNA was also digested with *Bam*HI or *Bgl*II to exclude the possibility of viral genomic rearrangement (Figure S4C). Viruses were then used to infect NIH3T3 or BHK21 cells to determine replication kinetics.

### Antibody production.

His-tagged vMAP (1–135) was purified from E. coli strain BL21 using Ni-conjugated agarose according to manufacturer's instructions (Qiagen). Urea was removed using Centricon (Millipore), after which protein concentration was measured with the Bradford method (Bio-Rad). Polyclonal antibodies against His-tagged vMAP (1–135) were produced in two rabbits.

### Plasmids.

Unless specified, all constructs were derived from pcDNA5/FRT/TO (Invitrogen) or pEF-IRES-puro [44]. A DNA fragment corresponding to the γHV-68 vMAP coding sequence was amplified from S11 genomic DNA by PCR and cloned into pcDNA5/FRT/TO between *Bam*HI and *Xho*I. To generate GFP fusion proteins, vMAP or vMAP (1–49) and its derivatives were PCR amplified and ligated to pEGFPN1 digested with *Bgl*II and *Sal*I. Mutations in the vMAP gene were generated by PCR using oligonucleotide-directed mutagenesis. All constructs were sequenced with an ABI PRISM 377 automatic DNA Sequenzer. Constructs expressing the HA-tagged and GFP-tagged Bcl-2 family proteins were kindly provided by J. Marie Hardwick (John Hopkins University), Beth Levine (UT-Southwestern Medical Center), and Richard J. Youle (National Institutes of Health), respectively. HA-Bcl-x_L_ and Flag-Bax were amplified by two-step PCR using GFP-Bcl-x_L_ and GFP-Bax as templates and cloned into pcDNA5/FRT/TO between the *Bam*HI and *Xho*I sites. Bcl-2 was cloned into pEYFP-C1 at the *Bam*HI site, and Bax and Bid were cloned into pECFP-C1 between the *Bam*HI/*Xho*I sites, respectively. To produce His-tagged vMAP protein from E. coli, vMAP (1–135) was PCR amplified and cloned into pQE40 (Qiagen). To express GST fusion proteins in mammalian cells, vMAP sequences were PCR amplified and cloned into pDEF3 digested with *Kpn*I and *Eco*RV.

### Immunoblotting and immunoprecipitation.

For immunoblotting, polypeptides were resolved by SDS-PAGE and transferred to PVDF membranes (Bio-Rad). Immunoblot detection was performed with anti-V5 (1:5000; Invitrogen), M2 anti-Flag (1:5000; Sigma), anti-GST (1:2000; Santa Cruz Biotechnology), anti-VDAC (porin 31HL) (1:1000; CALBIOCHEM), anti-Bax (6A7, 1:1000, Pharmingen), anti-COX4 (1:100; Clontech), anti-Bcl-2 (1:100, Santa Cruz Biotechnology), anti-Bcl-x_L_ (1:100, Pharmingen), or anti–cytochrome c (1:200, Clontech; 1:1000, Pharmingen). Rabbit anti-vMAP serum was diluted 1:3000 for immunoblots. Proteins were visualized using a chemiluminescence detection reagent (Pierce) and quantified using a Fuji Phosphor Imager.

For immunoprecipitation, cells were harvested and then lysed with 1% CHAPS buffer (Cell Signaling) supplemented with 1 mM dithiothreitol (DTT) and protease inhibitor cocktail (Roche). After pre-clearing with protein A/G–agarose beads for 1 h at 4 °C, whole-cell lysates were used for immunoprecipitation. Generally, 1–4 μg of commercial antibody or 1 μl of vMAP antiserum was added to 1 ml cell lysate, which was incubated at 4 °C for 8 to 12 h. After addition of protein A/G–agarose beads, the incubation was further extended for 1 h. The beads were extensively washed with lysis buffer, and the immunoprotein precipitates were eluted with SDS loading buffer by boiling for 5 min.

### Immunofluorescence microscopy.

Sixteen hours after transfection or electroporation, cells were fixed with 4% paraformaldehyde for 15 min, permeabilized with 0.2% (v/v) triton X-100 for 15 min, blocked with 10% goat serum in PBS for 30 min, and reacted with diluted primary antibody in PBS for up to 2 h at room temperature. After incubation, cells were washed extensively with PBS, incubated with the appropriate secondary antibody diluted in PBS for 30 min at room temperature, and washed three times with PBS. Mitochondrial staining was performed with 250 nM MitoTracker (Molecular Probes) for 20 min followed by washing with PBS at room temperature for 5 min. Alternatively, mitochondria were visualized using antibodies against mitochondrial proteins including cytochrome c and HSP60. Confocal microscopy was performed using a Leica TCS SP laser-scanning microscope (Leica Microsystems) fitted with a 100× Leica objective (PL APO, 1.4NA) and Leica imaging software. Images were collected at 512 × 512-pixel resolution. The stained cells were optically sectioned in the *z*-axis, and the images in the different channels (photo multiplier tubes) were collected simultaneously. The step size in the *z*-axis varied from 0.2 to 0.5 μm to obtain 16 slices per imaged file. The images were transferred to a Macintosh G4 computer (Apple Computer) and Photoshop (Adobe) was used to render the images.

For immunofluorescence microscopy, antibodies were directed against cytochrome c (1:200), Bax 6A7 (1:100; Pharmingen), HSP60 (1:100; Santa Cruz Biotechnology), vMAP (1:100), V5 (1:100; Invitrogen), or VDAC 31HL (1:100; CALBIOCHEM). All conjugated secondary antibodies were obtained from Molecular Probes and diluted at 1:1000 or 1:500. These included Alexa 488–conjugated goat anti–rabbit IgG, Alexa 568–conjugated goat anti–mouse IgG, Alexa 488–conjugated goat anti–mouse IgG, Alexa 594–conjugated donkey anti–goat IgG, Alexa 488–conjugated donkey anti–rabbit IgG, and Alexa 594–conjugated donkey anti–mouse IgG.

### GST pull-down.

In vitro GST pull-down was similar as previously described [45]. Briefly, E. coli (BL21) cells were induced with IPTG (1 μM) for 2 h and lysed with PBS containing 0.1% sarcosyl. GST or GST fusions were purified with glutathione-conjugated Sepharose beads and either used for immediate experiments or stored at −20 °C for future experiments. Then, whole-cell lysates were mixed with loaded glutathione-conjugated Sepharose and binding was extended at 4 °C for up to 2 h. After extensive washing, protein precipitates were resolved by SDS-PAGE and transferred to PVDF membrane, followed by immunoblotting.

For mammalian GST pull-down, 293T cells expressing GST fusion proteins and Bcl-2 family proteins were harvested and lysed with 1% CHAPS buffer (50 mM HEPES [pH 7.4]; 100 mM NaCl; 10 mM Tris; 1 mM EDTA; 1% CHAPS) supplemented with protease inhibitor cocktail (Roche). Supernatants after centrifugation procedures were pre-cleared with 15 μl of protein A/G beads at 4 °C for 1 h, after which 40 μl of 50% glutathione-conjugated Sepharose beads was added and binding was extended for 2 to 3 h at 4 °C. Protein precipitates were washed extensively with lysis buffer and analyzed by immunoblotting.

### Yeast two-hybrid screen.

The yeast two-hybrid screen was performed as previously described [45,46]. Yeast strain AH109 bearing the Gal4-vMAP (50–157) fusion gene plasmid was used to screen a cDNA library generated from EBV-immortalized B cells.

### Mitochondrial enrichment.

To obtain the mitochondrion-enriched HM fraction, 293T or NIH3T3 cells were harvested and washed with ice-cold PBS and resuspended with hypotonic buffer (10 mM Tris; 250 μM sucrose; 20 mM HEPES [pH 7.4]; 0.2 mM EDTA) supplemented with 1 mM DTT and protease inhibitor cocktail. The suspension was incubated on ice for 15 min and lysed with Nitrogen Bomber (Parr Instrument Company) at 250 psi for 15 min. Nuclei and unbroken cells were removed by centrifugation at 700*g* for 10 min. The supernatant at this point was regarded as whole cell lysate and was subjected to centrifugation at 6,000*g* for 15 min. The pellet was then resuspended with hypotonic buffer and centrifuged at 6,000*g* for 15 min; this process was repeated twice to obtain the mitochondrion-enriched fraction. The supernatant was further centrifuged at 100,000*g* for 90 min to yield the cytosolic fraction (supernatant), and the pellet was collected and regarded as the light membrane fraction. When cytochrome c release was examined, the step to obtain light membranes was skipped.

### Apoptosis assay.

Stably transfected NIH3T3 cells were grown in complete DMEM with 2 μg/ml puromycin. Cells, 1 × 10^6^ per well, were used for apoptosis induction with 1 μM ST for up to 16 h. After treatment, cells were harvested with cell dissociation buffer (Sigma) and fixed with 70% ethanol overnight. After staining the cells with PI for 30 min at room temperature, DNA content was measured with flow cytometry and analyzed with CellQuest (BD Biosciences). Alternatively, cells were treated with other apoptogenic agents (TNF-α plus cycloheximide, VESICULAR STOMATITIS infection, nocodazole), and sub-G1 cells were measured as described above.

For cytochrome c release, NIH3T3 cells were induced with 1 μM of ST for 4 h and harvested. The mitochondrion-enriched fraction was obtained as described above. Cytochrome c and mitochondrial membrane marker COX4 were detected by immunoblotting using the antibody from the ApoAlert kit (Clontech). For Bax mitochondrial translocation, cells were stimulated with 1 μM ST for 4 h and processed for intracellular fractionation as described above. For Bax activation, cells were stimulated with 1 μM ST for 4 h, and whole-cell lysates in 1% CHAPS buffer were precipitated with the mouse 6A7 monoclonal antibody or the rabbit P-19 polyclonal antibody, and protein precipitates were analyzed by immunoblotting.

For γHV-68ΔK3-GFP infection in wt and Bax^−/−^Bak^−/−^ DKO MEF cells, the activated caspase 3 was measured by intracellular staining according to manufacturer's instruction (BD Pharmingen), followed by flow cytometry analysis. Meanwhile, dead cells were stained with trypan blue and counted under light microscope.

### In vitro mitochondrion recruitment of Bcl-2.

Bcl-2 was in vitro translated from TNT-coupled reticulocyte lysate systems (Promega) and labeled with [^35^S]-methionine/cysteine. The mitochondrion-enriched HM fraction was obtained as described above from NIH3T3/puro or NIH3T3/vMAP cells. Protein was measured with the Bradford method and 50 μg of mitochondrial proteins were mixed with 100 μl of reticulocyte lysates containing the radioactively labeled Bcl-2. The mixtures were incubated at 30 °C for 2 h and mitochondria were pelleted at 13,000 rpm for 15 min. While the supernatants were collected as membrane-free Bcl-2, the pellets were washed twice with hypotonic buffer and resuspended in PBS as mitochondrion-associated Bcl-2. Proteins were then analyzed by gradient (4%–12%) SDS-PAGE and Phosphor Image reader.

### Fluoresence resonance energy transfer (FRET) assay.

NIH3T3/vec, NIH3T3/vMAP, and NIH3T3/vMAPΔ20 were transfected with pEYFP-Bcl-2 and pECFP-Bid. At 36 h post-transfection, cells were harvested with dissociation buffer and washed twice with room temperature PBS containing 0.5% bovine serum albumin. Next, cells were subjected to flow cytometry that is equipped with fluorochrome excitation capability.

## Supporting Information

Figure S1 The Mitochondrion-Dependent Apoptosis Limits γHV-68 Replication (A) γHV-68 replication in wild-type (wt) or Bax^−/−^Bak^−/−^ (DKO) MEFs. wt or DKO MEFs were infected with γHV-68ΔK3-GFP at a multiplicity of infection (MOI) of 1, and subsequently photographed at post-infection day 1 (10×) and day 2 (4× objective).
(B) Replication kinetics of γHV-68ΔK3-GFP. wt and DKO MEFs were infected with γHV-68ΔK3-GFP at an MOI of 1. Cells and supernatants were harvested at various time points. Virus titer was determined by plaque assay in BHK21 cells. Data represent the result of four independent measurements and error bars indicate standard deviation with (*) *p* < 0.05 relative to wt MEF cells as calculated by Student's *t*-test.
(C) Intracellular activated caspase 3 staining. wt or DKO MEFs were infected with γHV-68ΔK3-GFP and subsequently harvested at various time points. Cells were stained with PE-conjugated antibody that is specific for the activated caspase 3 and analyzed by flow cytometry. Data represent one of two independent experiments.
(D) Trypan blue staining upon γHV-68ΔK3-GFP infection. Viral infection was carried out as described in (C). wt or DKO MEFs were harvested at day 3 post-infection and stained with trypan blue. Data represent the average of two independent experiments and error bars indicate standard deviation with (*) *p* = 0.02 relative to wt MEF cells as calculated by Student's *t*-test.
(428 KB TIF)

Figure S2 Mitochondrial Localization of vMAP (A) The reactivity of anti-vMAP serum. COS-1 cells expressing vMAP-V5 were fixed and stained with rabbit pre-immune serum or anti-vMAP serum. A single representative optical section is presented.
(B) The mitochondrial localization of vMAP-GFP fusion protein by subcellular fractionation. NIH3T3 cells were transiently transfected with pEGFP-N1 plasmid or its derivatives containing vMAP (1–40) or vMAP (1–30). At 36 h post-transfection, cells were harvested and subjected to fractionation. Twenty μg of proteins were analyzed by immunoblotting to detect GFP fusion proteins with a rabbit anti-GFP antibody (top two panels). Immunoblotting with anti-COX4 and anti-actin antibodies were included as controls (bottom two panels). WCL, whole-cell lysate; HM, mitochondrion-enriched heavy membrane; Cyto, cytosolic fraction.
(C) vMAP localizes to the mitochondria. NIH3T3 cells were transfected with pcDNA5-vMAP. At 16 h post-transfection, cells were fixed and stained with anti-Hsp60 antibody, anti-cytochrome c antibody, or anti-vMAP serum. A single representative optical section is presented.
(D) vMAPΔ20 localizes to the mitochondrion. COS-1 cells transfected with vMAPΔ20 expression vector. At 16 h post-transfection, cells were fixed and stained with mouse anti-cytochrome c antibody (green) and rabbit anti-vMAP serum (red). A single representative optical section is presented.
(E) The expression of vMAP and its mutants in NIH3T3 stable cell lines. Twenty μg of lysates of NIH3T3/puro, NIH3T3/vMAP (wt), NIH3T3/vMAPΔ20 (Δ20), NIH3T3/vMAP L/A (L/A), or NIH3T3/vMAPΔ20L/A (Δ20L/A) cells were analyzed by immunoblotting with rabbit anti-vMAP serum (top panel) or anti-actin antibody (bottom panel).
(255 KB TIF)

Figure S3 Characterization of vMAP Interaction with Bcl-2 and Bcl-x_L_
(A) vMAP interacts with Bcl-2 in 293T cells. Lysates from 293T cells expressing vMAP and/or HA-Bcl-2 were immunoprecipitated with anti-HA (Bcl-2) and analyzed by immunoblotting with anti-vMAP serum (top panel). WCLs were analyzed by immunoblotting with anti-vMAP serum (middle panel) and anti-HA antibody (bottom panel).
(B) vMAP interacts with the hydrophobic core of Bcl-2. Lysates of 293T cells transfected with HA-Bcl-2_85–186_ together with GST (lane 2) or vMAP(1–50)-GST (lane 3) were used for GST pull-down, followed by immunoblotting with anti-HA (Bcl-2, top panel). WCLs were analyzed by immunoblotting with anti-HA (Bcl-2, middle panel) or anti-GST (bottom panel) antibodies. Lane 1 indicates 2% of input for GST pull-down.
(C) Bcl-2 interaction with Bid. At 48 h post-transfection with HA-Bcl-2, HA-Bcl-2 G_145_A, or HA-Bcl-2 W_188_A expression vector together with GST (lane 2) or GST-Bid (lane 3) expression vector, 293T cell lysates were used for GST pull-down, followed by immunoblotting with anti-HA (Bcl-2, top panel). WCLs were analyzed by immunoblotting with anti-HA (Bcl-2, middle panels) and anti-GST (bottom panels), respectively. Lane 1 indicates 2% of input for GST pull-down.
(D) vMAP does not affect the Bcl-x_L_ interaction with Bax. NIH3T3/puro (lanes 2 and 3) or NIH3T3/vMAP (lane 4) cells were transfected with HA-Bcl-x_L_ and Flag-Bax expression vectors. WCLs were used for immunoprecipitation with anti-HA or anti-Myc (control), followed by immunoblotting with anti-Flag (Bax). WCLs were analyzed by immunoblotting with anti-HA (Bcl-x_L_, middle) and anti-Flag (Bax, bottom panel) antibodies. Lane 1, 5% of input from NIH3T3/puro cells. Anti-Myc antibody was included as a negative control.
(E) vMAP does not affect the interaction between Bcl-2 and Bax/Bak. NIH3T3/puro (lanes 1, 2, 4, and 5) and NIH3T3/vMAP (lanes 3 and 6) cells were transfected with Flag-Bcl-2 and HA-Bax or HA-Bak. Immunoprecipitation was carried out as described in Figure S3D. Anti-Myc antibody was included as a negative control for immunoprecipitation. Protein precipitates were analyzed by immunoblotting with HRP-conjugated anti-Flag to detect Flag-Bcl-2. WCLs were analyzed by immunoblotting with anti-HA antibody (HA-Bax or HA-Bak, middle panels) and anti-Flag antibody (Flag-Bcl-2, bottom panel).
(F) FRET assay demonstrates that vMAP does not affect the Bcl-2 interaction with Bax. At 36 h post-transfection with pEYFP-Bcl-2 and pECFP-Bax, NIH3T3/puro cells (Puro), NIH3T3/vMAP cells (vMAP), and NIH3T3/vMAPΔ20 cells (vMAPΔ20) were harvested and subjected to flow cytometry analysis. Numbers in the FRET diagrams indicate the percentage of the transfected cell population without protein interaction (lower right) and the transfected cell population with protein interaction (upper left). Data represent results from two independent experiments.
(334 KB TIF)

Figure S4 Generation of γHV-68 ΔvMAP and Its Revertant (A) Schematic illustrations of γHV-68 wt, γHV-68 ΔvMAP Kan^R^, and γHV-68 ΔvMAP. Specific mutations include T > A changes at the nucleotide positions of 76016, 76049, and 76076, and a G > A change at the nucleotide position of 76079 (Entrez accession number U97553).
(B) Detection of vMAP expression in γHV-68 wt (wt), γHV-68 ΔvMAP (ΔvMAP), or γHV-68 Rev (Rev) infected NIH3T3 cells. NIH3T3 cells were infected with these recombinant γHV-68 at MOI = 1 and cells were harvested at 16 h post-infection. Cell lysates were used for immunoprecipitation with anti-vMAP serum, followed by immunoblotting with anti-vMAP serum. Arrow indicates vMAP protein.
(C) Restriction digestion of wt and mutant γHV-68 genomic DNAs. Genomic DNAs from γHV-68 wt (lane 1), γHV-68 ΔvMAP (lane 2), or γHV-68 Rev (lane 3) were purified by alkaline lysis and ethanol precipitation, digested with *Bgl*II and *Bam*HI, resolved on 0.7% agarose gel, and photographed.
(161 KB TIF)

Figure S5 vMAP Does Not Affect the Bcl-2 with VDAC1 Interaction Lysates of NIH3T3/puro (lane 1), NIH3T3/vMAP (lane 2), and NIH3T3/vMAPΔ20 (lane 3) cells were immunoprecipitated with a normal rabbit serum or an anti-Bcl-2 rabbit antibody. Protein precipitates were analyzed by immunoblotting with antibodies to VDAC1 (top panel), Bcl-2 (middle panel), and anti-vMAP serum (third panel from top). WCLs were analyzed by immunoblotting with anti-vMAP serum (bottom panel). H, the heavy chain of IgG.
(164 KB TIF)

### Accession Numbers

The Entrez Nucleotide (http://www.ncbi.nlm.nih.gov/sites/entrez?db=Nucleotide) accession numbers for the sequences discussed in this paper are vMAP (U97553); human Bcl-2 (NM_000633); human Bcl-x_L_ (NM_138578); mouse Bax (BC053380); human Bak (U16811); mouse Bid (U75506); mouse Bad (L37296).

## Abbreviations

γHV-68: murine γ-herpesvirus 68
ANT: adenine nucleotide translocator
BH: Bcl-2 homology
DKO: double knockout
FRET: fluorescence resonance energy transfer
HM: heavy membrane
LM: light membrane
MEF: murine embryonic fibroblast
MTS: mitochondrial targeting sequence
ORF: open reading frame
PI: propidium iodide
PTP: permeability transition pore
ST: staurosporine
VDAC1: voltage-dependent anion channel 1
vMAP: viral mitochondrial anti-apoptotic protein
wt: wild-type
